# The transcriptome of the Bermuda fireworm *Odontosyllis enopla* (Annelida: Syllidae): A unique luciferase gene family and putative epitoky-related genes

**DOI:** 10.1371/journal.pone.0200944

**Published:** 2018-08-08

**Authors:** Mercer R. Brugler, M. Teresa Aguado, Michael Tessler, Mark E. Siddall

**Affiliations:** 1 Division of Invertebrate Zoology, American Museum of Natural History, New York, New York, United States of America; 2 Biological Sciences Department, NYC College of Technology, City University of New York, Brooklyn, New York, United States of America; 3 Departamento de Biología, Universidad Autónoma de Madrid, Cantoblanco, Madrid, Spain; Evergreen State College, UNITED STATES

## Abstract

The Bermuda fireworm *Odontosyllis enopla* exhibits an extremely tight circalunar circadian behavior that results in an impressive bioluminescent mating swarm, thought to be due to a conventional luciferase-mediated oxidation of a light-emitting luciferin. In addition, the four eyes become hypertrophied and heavily pigmented, and the nephridial system is modified to store and release gametes and associated secretions. In an effort to elucidate transcripts related to bioluminescence, circadian or circalunar periodicity, as well as epitoky-related changes of the eyes and nephridial system, we examined the transcriptomic profile of three female *O*. *enopla* during a bioluminescent swarm in Ferry Reach, Bermuda. Using the well-characterized luciferase gene of the Japanese syllid *Odontosyllis undecimdonta* as a reference, a complete best-matching luciferase open reading frame (329 amino acids in length) was found in all three individuals analyzed in addition to numerous other paralogous sequences in this new gene family. No photoproteins were detected. We also recovered a predicted homolog of 4-coumarate-CoA ligase (268 amino acids in length) that best matched luciferase of the firefly *Luciola* with the best predicted template being the crystal structure of luciferase for *Photinus pyralis*, the common eastern firefly. A wide variety of genes associated with periodicity were recovered including predicted homologs of *clock*, *bmal*1, *period*, and *timeless*. Several genes corresponding to putative epitoky-related changes of the eyes were recovered including predicted homologs of a phototransduction gene, a retinol dehydrogenase and carotenoid isomerooxygenase as well as a visual perception related retinal rod rhodopsin-sensitive cGMP 3',5'-cyclic phosphodiesterase. Genes correlating to putative epitoky-related changes of the nephridia included predicted homologs of nephrocystin-3 and an egg-release sex peptide receptor.

## Introduction

**Fig 1:**
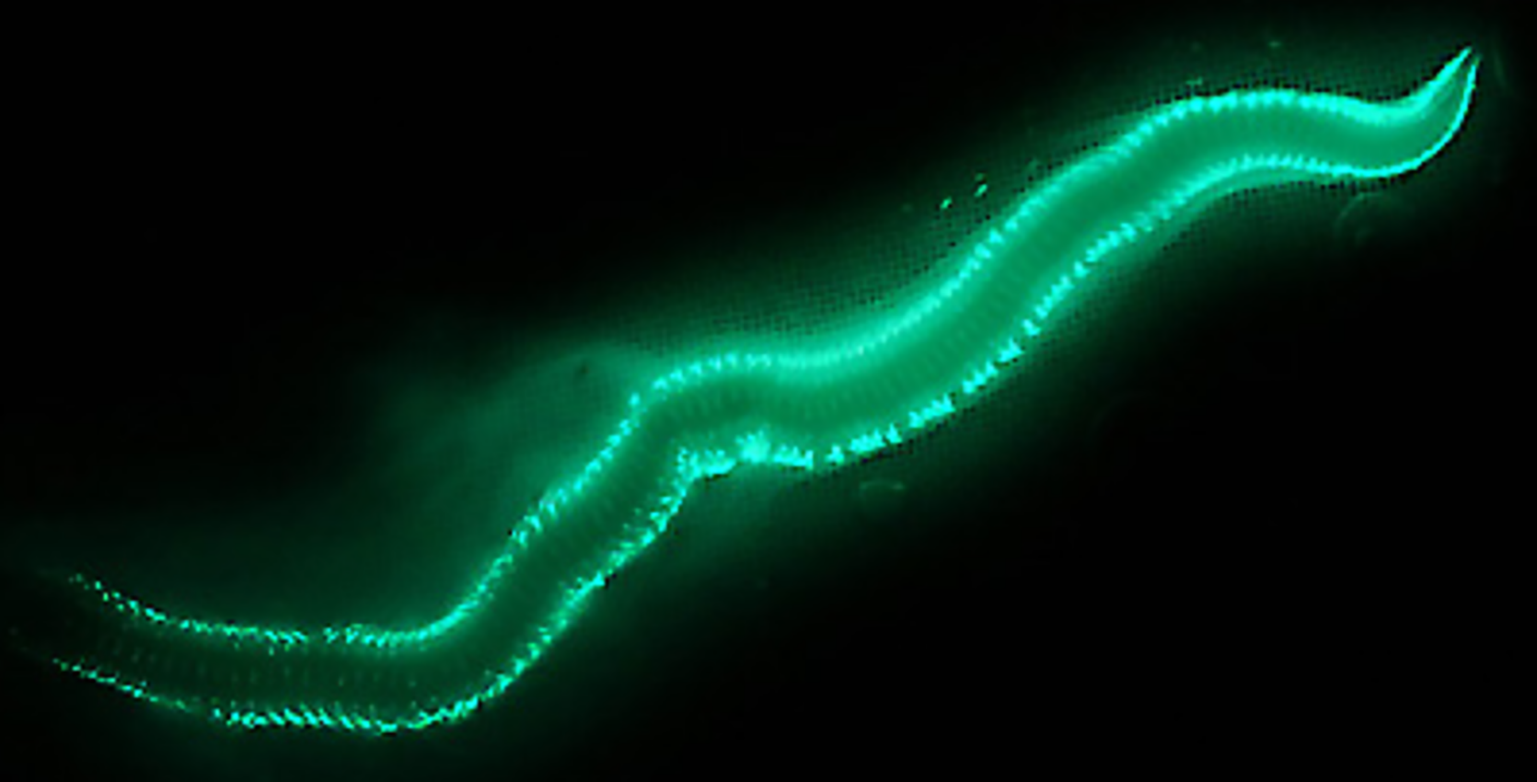
Bioluminescent display of *Odontosyllis enopla*. During the breeding period, female *Odontosyllis enopla* swim in slow circles secreting a bright bluish-green luminous mucus while releasing gametes. Photo credit: Dr. James B. Wood.

On the night of October 11, 1492, lights were seen from the stern deck of *La Santa María* by Christopher Columbus himself and his crew just in advance of their historic landfall at El Salvador Island, currently also known as Watling Island in the Bahamian archipelago [1]. The lights were described as “the flame of a small candle alternately raised and lowered,” “some distance away in the darkness,” and “gone out of sight again long after.” While mysterious and left unexplained at the time of one of the most monumental events in recent human history, these lights later found plausible explanation. The description of the event, the geographic location and timing, as well as duration match the monthly bioluminescent reproductive swarms of polychaetes in the genus *Odontosyllis* (Fig 1) [1].

*Odontosyllis* species periodically, and in accordance with a lunar cycle, leave the benthos, become epitokous (*i*.*e*., an egg- or sperm-carrying body) and show an impressive bioluminescent display [2, 3, 4]. Swarming episodes during summer and early autumn months occur on the first nights after the full moon with highly predictable timing [2, 3]. The events begin approximately one hour after astronomical sunset and continue for 10–30 minutes [2, 3, 4, 5, 6]. Lunar cycles are oftentimes supplemented with interindividual communication in the form of chemical (*i*.*e*., pheromone) and/or visual cues [4]. Spawning females swim in slow circles secreting a bright bluish-green luminous mucus while releasing gametes [3, 7, 8, 9]. Males swim rapidly toward glowing females while emitting short flashes of light in advance of releasing their own gametes [5]. Epitoky is reversible and the animals often survive after the mating ritual for further reproductive activity [10].

Reproductive individuals of *Odontosyllis* not only undergo physiological and behavioral modifications, but metamorphosis as well [4]. This includes modification of the appendages (*i*.*e*., parapodia), which includes specialized chaetae for swimming close to the surface, as well as the eyes; the four prostomial eyes of both sexes become hypertrophied, although this change is more apparent in males [4]. During reproductive periods, the eyes of females also become heavily pigmented with a carotenoid (putatively a rhodopsin system), with the anterior eyes becoming much larger than the posterior eyes [7]. Additionally, the nephridia are modified to store and release gametes, as well as release fluids that provide nutrition and support for the gametes [4].

*Odontosyllis* species are classified in the annelid family Syllidae, which contains 74 genera and 700+ species [11, 12]. *Odontosyllis* is currently a member of the subfamily Eusyllinae, reorganized to be monophyletic by Aguado et al. [13]. The genus comprises 55 species (WoRMS), though phylogenetic analyses that included several species revealed that it may not be a monophyletic group [13, 14]. The bioluminescent genera within the Syllidae are *Odontosyllis*, *Eusyllis*, and *Nudisyllis*, the three with a relatively large number of species. For *Odontosyllis*, several species have been documented with light emitting properties, and within *Eusyllis* and *Nudisyllis* at least one species, respectively (i.e., *E*. *blomstrandi* and *N*. *pulligera*) [15, 16]. It has been suggested that this species-level diversity could be attributed to strong sexual selection stemming from bioluminescent courtship displays [17]. The Syllidae dominate many benthic communities and are known for a striking variety of reproductive modes [4, 13, 14, 18]. Bioluminescence also occurs in several phylogenetically diverse marine and terrestrial annelid lineages [19, 20, 21]. *Chaetopterus* and *Mesochaetopterus* and the terebellid *Polycirrus* release a luminescent glow when disturbed [22, 23]. Some species of *Tomopteris* produce light of different colors [24] and a group of deep-sea polychaetes releases green luminescent “bombs” when alarmed (the “bombs” are fluid-filled structures, homologous to segmental branchiae, that emit light when released [25]). Whereas several polynoids use a protein triggered with superoxide radicals [26], the biochemical mechanisms related to bioluminescence are not yet well understood for other taxa [27].

Bioluminescence in the “Bermuda fireworm” *Odontosyllis enopla* has been the subject of considerable study [2, 3, 5, 7, 28, 29, 30] and is thought to be due to a conventional luciferase-mediated oxidation of a light-emitting luciferin [31]; though some have speculated on the involvement of a secondary photoprotein [9, 32]. Support for a luciferin-based system comes from [7, 30] which showed that the eyes of males, which are larger than females, display increased spectral sensitivity (~510–520 nm) in the luciferin emission spectrum (507–516 nm).

Other species of *Odontosyllis* that have been documented as bioluminescent include: *O*. *phosphorea* [6, 9], *O*. *luminosa* [8], *O*. *octodentata* [33], *O*. *polycera* [10], and *O*. *undecimdonta* [34, 35], among others. The mysterious lights described by Columbus have been attributed to *O*. *enopla* or *O*. *luminosa* [1, 3, 8]. While bioluminescence in these polychaetes is directly related to intraspecific reproductive behavior, it might also be a multifunctional process [9]. For example, luminescence during non-swarming periods has been documented for the benthic forms of *O*. *enopla* and *O*. *phosphorea* in response to physical disturbance, which has been considered a possible deterrent strategy against predators [3, 9]. Moreover, Gaston and Hall [8] proposed that bioluminescence in *O*. *luminosa* is indeed used as an aposematic signal, since their predators were observed to regurgitate recently ingested luminescent worms. *Eusyllis blomstrandi* is also known to emit light not only for reproductive purposes but also as defensive mechanisms. They are able to detach their posterior luminescent part of their bodies to distract predators while the anterior end escapes [15].

During the last decade, new technologies and progress in sequencing techniques have made it possible to elucidate whole genomes and transcriptomes [36]. Transcriptome sequencing has been applied widely for different purposes in annelid research; e.g., to reconstruct the Annelida Tree of Life [37] or to find certain genes that participate in specific biological processes, such as adaptation to deep sea and extreme environments [36], various larval developmental modes [38], anticoagulant capabilities [39], reproductive processes [40], and characterization of certain venomous toxins [41]. Recently, Mehr et al. [42] found several genes in *Hermodice carunculata* (Amphinomidae) that could be involved in light production, though the species is not known to be bioluminescent. The first available syllid transcriptome was provided by Weigert et al. [37], and recently, the transcriptome of *Typosyllis antoni* has been presented as a tool for the study of developmental and evolutionary processes in the Syllidae (Ponz et al., *submitted* [43]). Schultz et al. [44] used a combination of bioluminescent protein purification, luciferin purification, *in vitro* expression, and RNA sequencing to identify and characterize a luciferase within the Japanese syllid *O*. *undecimdonta*. The authors concluded that the luciferase of *O*. *undecimdonta* is “evolutionarily unique” as they found no identifiable homologous proteins when querying publicly available datasets using BLAST and HMMER.

Herein we examine the transcriptomic profile of female *O*. *enopla* during a bioluminescent swarm in an effort to elucidate transcripts related to bioluminescence, circadian and circalunar periodicity, as well as changes of the eyes and nephridial system. Genes involved in bioluminescence processes are not only interesting from an evolutionary point of view but also because of their possible biotechnological applications [45].

## Materials & methods

After acquiring a collection permit (#151001) from the Bermuda Department of Environmental Protection, we collected 12 female *Odontosyllis enopla* from Ferry Reach, St. George’s Parish, Bermuda (32.362368, -64.714034) on October 30, 2015 beginning at 7:26 pm (the third night after the full moon, 55 minutes after sunset and 90 minutes after a -3.7 cm low tide). Bioluminescing female worms were captured from the water surface with live insect forceps (Fine Science Tools, Foster City, CA, USA) and immediately immersed in 40 mL of RNAlater (Thermo Fisher Scientific, Waltham, MA, USA). Specimens were individually placed in fresh RNAlater in cryotubes and stored at -80°C at the Bermuda Institute of Ocean Sciences prior to export to the American Museum of Natural History (New York City) in April 2016. Total RNA was isolated from the whole body of three of the 12 worms with a modified RNeasy Tissue Kit (Qiagen) protocol (see [39] for details). Final RNA concentration was determined with the Agilent RNA 6000 Nano Kit on an Agilent 2100 Bioanalyzer. Isolates were prepared using the TruSeq Stranded mRNA Library Prep Kit (Illumina, San Diego, CA) with a 350 bp insert size and run at the New York Genome Center on an Illumina HiSeq 2500 (2 x 125 bp) allocating one-eighth of a lane for each isolate.

Adaptor sequences, polyadenylation and low-quality regions (Phred score <20) of resulting reads were trimmed with Trimmomatic [46]. Overall quality of reads was verified with FastQC v0.10.1 and have been deposited in NCBI’s Short Read Archive under BioProject ID PRJNA448700. Filtered reads were assembled using the default parameters of the Trinity de novo assembler version r20130225 for each *O*. *enopla* specimen independently. Assembled contigs then were examined with Transdecoder v. 3.0.0. (https://transdecoder.github.io/) for best-predicted open reading frames (ORFs) greater than 100 amino acids in length. Assembled contigs (with blastx) and predicted ORFs (with blastp) were screened against published sequences for a recently described luciferase from *O*. *undecimdonta*, an additional 536 annotated luciferase proteins, 71 annotated photoproteins, and 23 annotated proteins pertaining to periodicity (*i*.*e*., *clock*, *period*, *timeless*, *bmal*, *cry*, *timeout*, *pdp1* and *vrille*; these stand-alone databases of known annotated luciferase and photoprotein genes are available upon request) and against 2748 KOGs (euKaryotic Orthologous Groups; comprised of 458 core eukaryotic genes for six species; [47]). Matching contigs and ORFs exceeding a threshold of 1e^-10^ from this screening were then compared to the UniProtKB/Swiss-Prot annotated database of NCBI. Sequences that did not return best reciprocal matches in this manner were not analyzed further. Conserved domain architecture and active site prediction were determined in relation to NCBI curated domains (CDD online, v3.16) [48]. The best scoring hit to the annotated database of luciferase proteins (i.e., an ORF 268 amino acids in length) was also blasted against the annotated transcriptome of *Typosyllis antoni* (Ponz et al., *submitted* [43]) to further confirm or refute its putative identity.

Illumina reads (150 x 150 bp) produced by Schultz et al. [44] were assembled de novo using default parameters in Trinity (v2.6.5) in order to further examine the extent of paralogs of the *Odontosyllis*-specific luciferase gene family.

Genes associated with putative epitoky-related changes of the eyes and nephridia were recovered via a blastp comparison of *O*. *enopla* transcripts (for all three individuals) against the entire nr database. Using default parameters, the RaptorX web server [49] was used to predict the protein structure and function of the *O*. *enopla* CoA ligase, which is 268 amino acids in length.

## Results

The Illumina HiSeq 2500 generated 37,063,191 (Individual #1), 39,513,743 (Individual #2), and 34,329,885 (Individual #3) raw reads. After trimming adaptors and low-quality regions, assembly with Trinity yielded 176,598 (Individual #1), 207,006 (Individual #2) and 283,041 (Individual #3) contigs (including splice variants) for the three female *Odontosyllis enopla* worms. These represented 44,426 (Individual #1), 49,458 (Individual #2) and 61,002 (Individual #3) open reading frames (>100 amino acids) predicted by Transdecoder, and included >99.0% of the 2,748 core KOGs. Missing from the expected core eukaryotic genetic repertoire for all three worms were neutral trehalase (carbohydrate transport and metabolism; KOG0602) and NAD/FAD-utilizing protein (possibly involved in translation; KOG2311).

**Fig 2:**
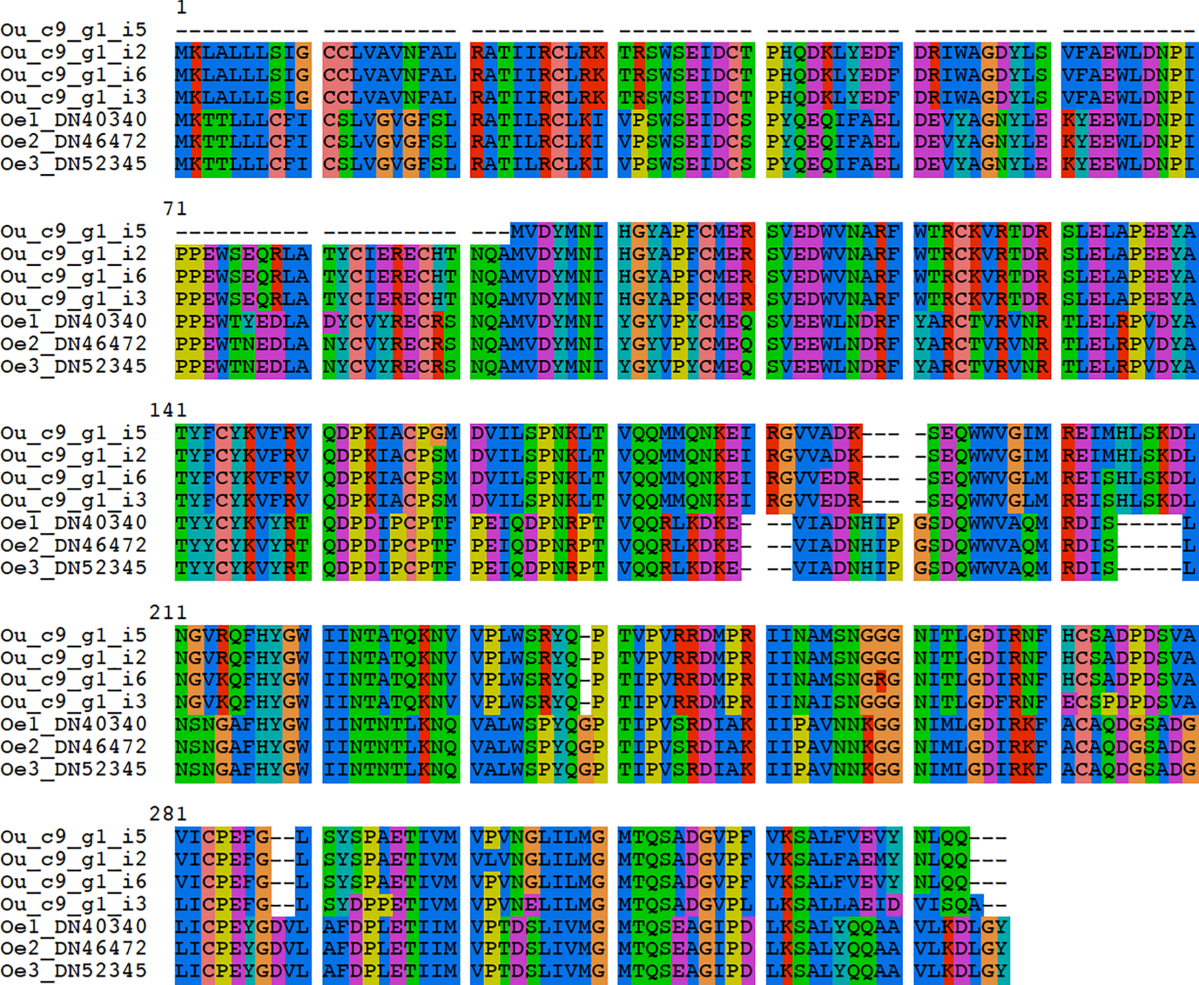
Multiple sequence alignment of the *Odontosyllis enopla* luciferase gene with that of the Japanese syllid *O*. *undecimdonta*. The *Odontosyllis enopla* luciferase gene (329 amino acids in length) is aligned with the four putative luciferase transcripts (isoforms) of *O*. *undecimdonta*. The alignment was generated using default parameters and the L-INS-i iterative refinement method within MAFFT (v7.402). Ou: *O*. *undecimdonta*. Oe1: *O*. *enopla* Individual 1.

Among contigs for each worm were (respectively) 7, 4 and 5 principal assembly isoform groups that matched (at 1e^-20^ to 1e^-107^) known luminescent proteins from *O*. *undecimdonta* (Fig 2), and an additional 483, 503 and 536 that yielded high scoring hits to a stand-alone database of other known annotated luciferase/monooxygenase genes.

Only 4, 2 and 1 yielded hits to the stand-alone database of known annotated photoprotein genes. Reciprocal comparison to UniProtKB/Swiss-Prot and the nr database yielded no other matching sequences for the transcripts matching the known luminescent proteins from *O*. *undecimdonta* and otherwise only yielded a single complete firefly luciferase-like open reading frame (268 amino acids in length) from two of the three worms (Conserved Protein Domain Family [hereby abbreviated CPDF]: *Firefly_Luc_like* [cd05911]) at 4.72e^-79^). No photoproteins resulted from reciprocal comparisons to UniProtKB/Swiss-Prot. The foregoing amino acid sequence produced no specific high-scoring matches to any other annelid sequences in the nr, RefSeq, or EST databases of NCBI. In the absence of functional data, we could not confirm the identity of this luciferase-like ORF as a true luciferase. Thus, in an effort to determine which superfamily this ORF is affiliated with, we conducted a blastp analysis against the annotated transcriptome of *Typosyllis antoni* (Ponz et al., *submitted* [43]), which resulted in a best hit to 4-coumarate-CoA ligase (e-value: 5e^-66^). The RaptorX web server predicted a single domain (with 100% of the 268 residues modeled) for the *O*. *enopla* CoA ligase, with the best template being the crystal structure of luciferase (RCSB Protein Data Bank template ID: 5DV9A) for *Photinus pyralis*, the common eastern firefly, at a p-value of 1.57e^-17^.

**Fig 3:**
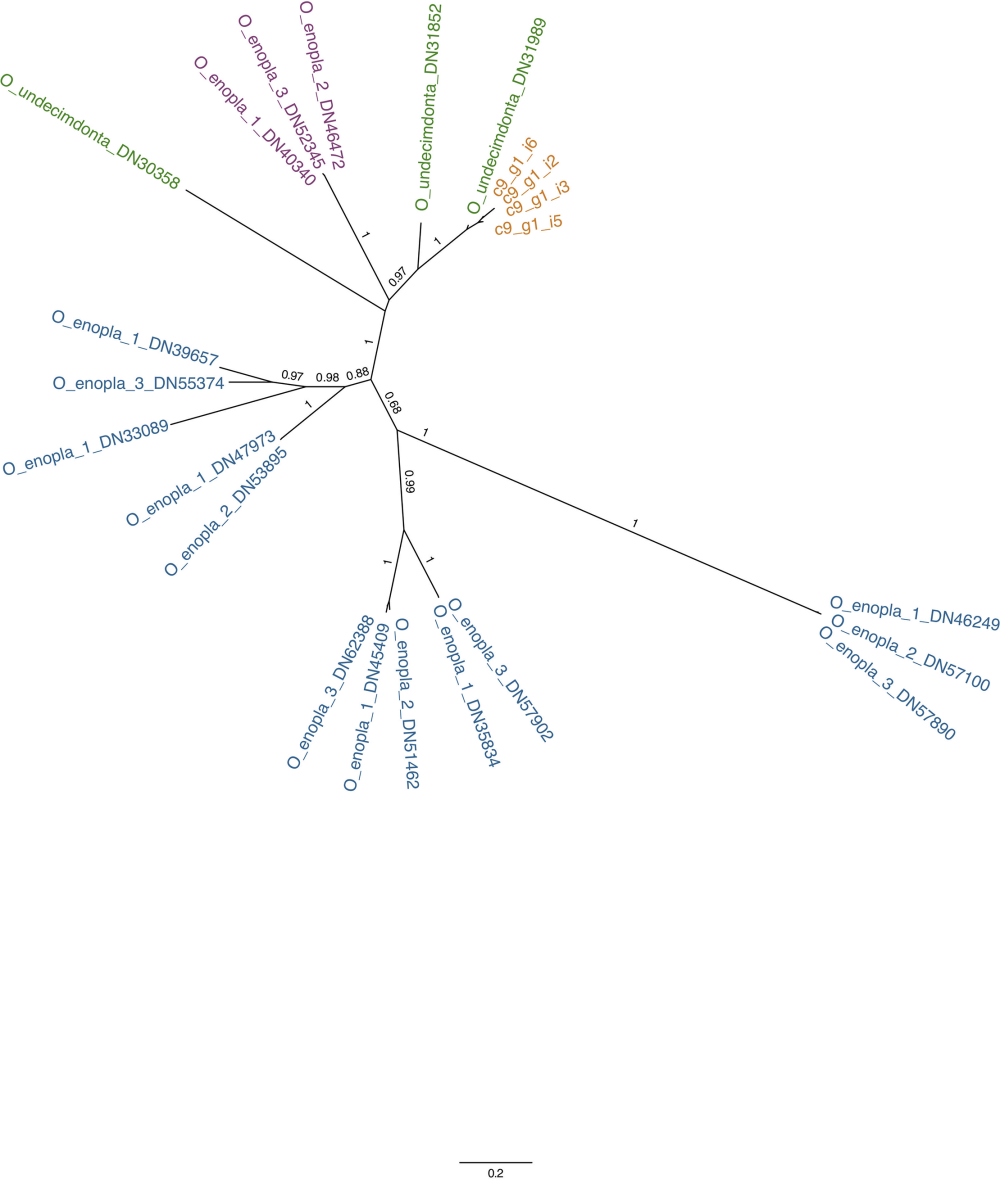
An unrooted maximum likelihood-based phylogenetic tree showing the relationship of both orthologs and paralogs of the luciferase gene for *Odontosyllis enopl*a and *O*. *undecimdonta*. The four transcripts (isoforms) found by Schultz et al. [44] are in orange. ‘O_undecimdonta_DN31989’ (green) is identical to one of the four isoforms but has a different name because it is based on our Trinity assembly. The two additional green terminals are paralogs of the *O*. *undecimdonta* luciferase that were not reported by Schultz et al. [44]. For *O*. *enopla*, orthologs are shown in purple and the paralogs in blue. O_enopla_1: Individual 1, O_enopla_2: Individual 2, O_enopla_3: Individual 3. The ML tree was constructed using a MUSCLE-based amino acid alignment and the following parameters: WAG + gamma + I model; aLRT-based support values.

The luciferase genes from individuals 2 and 3 of *O*. *enopla* were identical at the amino acid level; however, the luciferase of individual 1 contained two variable sites when compared to individuals 2 and 3 (p-distance: 0.608%). At the nucleotide level, the difference was 1.01% when comparing individual 1 with 2 and 3, and 0.606% when comparing individual 2 with 3. Given the number of paralogs of the luciferase gene found in *O*. *enopla*, we assembled the raw Illumina reads produced by Schultz et al. [44] and searched for paralogs of the luciferase gene in *O*. *undecimdonta*. We recovered two paralogs of the luciferase gene in *O*. *undecimdonta*; however, neither were reported by Schultz et al. [44] (Fig 3).

We compared each of the luciferase paralogs found within each *O*. *enopla* to the transcriptome of *O*. *undecimdonta* and did not find any significant matches.

A wide variety of genes putatively associated with periodicity were recovered from comparison of *O*. *enopla* transcripts against annotated local databases followed by reciprocal verification against UniProtKB/Swiss-Prot; these included predicted homologs of *clock* (GenBank Acc No AGX93013), *bmal*1 (GenBank Acc No O88529), *period* (GenBank Acc No AEJ87229), and *timeless* (GenBank Acc No AGX93010) (S1 Fig). In addition, two putative photosensitive cryptochrome transcripts were found corresponding to predicted homologs of the light-receptive (*l-cry*: GenBank Acc No AEJ87227) and transcriptional repressive (*tr-cry*: GenBank Acc No AGX93012) forms known from *Platynereis dumerilii* (S2 Fig) [50]. Orthologs representing *timeout*, *vrille*, or *pdp1* were not found in any of the three female *O*. *enopla*.

Eight genes associated with putative epitoky-related changes of the eyes (Table 1 and S3 Fig) and three genes associated with putative epitoky-related changes of the nephridia (Table 2 and S4 Fig) were recovered via a blastp comparison of *O*. *enopla* transcripts (for all three individuals) against the entire nr database.

**Table 1:** Genes associated with putative epitoky-related changes of the eyes in female *Odontosyllis enopla* (Results reported for Individual #1 only).

Predicted Homolog	Length (aa)	Top Hit / GenBank Accession Number	E-Value	Identities / Positives
Phototransduction (G-alpha protein)	354	Sabellid polychaete *Acromegalomma interruptum* / ASQ43263	0.0	343/354 (97%) / 350/354 (99%)
Retinal rod rhodopsin-sensitive cGMP 3',5'-cyclic phosphodiesterase subunit delta	147	Elephant shark *Callorhinchus milii* / NP_001279845	5e^-89^	121/144 (84%) / 135/144 (94%)
Phosphatidate cytidylyltransferase (photoreceptor)	442	Scallop *Mizuhopecten yessoensis* / XP_021375015	0.0	320/452 (71%) / 374/452 (83%)
Retinol dehydrogenase (retinaldehyde reductase)	327	Cephalochordate *Branchiostoma floridae* / XP_002605547	2e^-97^	162/329 (49%) / 216/329 (66%)
Epidermal retinol dehydrogenase 2-like isoform	306	Bivalve *Crassostrea virginica* / XP_022308172	1e^-136^	182/303 (60%) / 233/303 (77%)
Retinitis pigmentosa GTPase regulator	761	Polyplacophoran *Leptochiton asellus* / ASM47589	0.0	286/490 (58%) / 365/490 (74%)
Carotenoid isomerooxygenase-like gene	529	Bivalve *Mizuhopecten yessoensis* / XP_021374132	2e^-115^	190/503 (38%) / 288/503 (57%)
Class A rhodopsin-like G-protein coupled receptor	319	Insect *Pediculus humanus corporis* / XP_002431639	2e^-11^	67/274 (24%) / 121/274 (44%)

**Table 2:** Genes associated with putative epitoky-related changes of the nephridia in female *Odontosyllis enopla* (Results reported for Individual #1 only).

Predicted Homolog	Length (aa)	Top Hit / GenBank Accession Number	E-Value	Identities / Positives
Nephrocystin-3	347	Bivalve *Crassostrea virginica* / EKC34119	4e^-149^	222/325 (68%) / 273/325 (84%)
Nephrocystin-3-like gene	437	Bivalve *Mizuhopecten yessoensis* / XP_021351806	3e^-148^	228/436 (52%) / 301/436 (69%)
Sex peptide receptor-like gene	178	Bivalve *Crassostrea virginica* / XP_022289073	3e^-68^	101/164 (62%) / 124/164 (76%)

All 11 genes associated with putative epitoky-related changes of the eyes and nephridia were found in Individuals #1 and #2, while 10 of the 11 genes were found in Individual #3 (a transcript for a predicted homolog of nephrocystin-3 was not located).

## Discussion

A complete luciferase open reading frame was found in all three individuals of *Odontosyllis enopla* that were analyzed which were highly similar to the expressed luciferase gene of the Japanese syllid *O*. *undecimdonta* (Schultz et al. [44]). These luciferase genes and their paralogs are evolutionarily unique; like Schultz et al. [44], we found no other identifiable homologous proteins when querying publicly available databases. No photoproteins were detected in any of the *O*. *enopla* transcriptomes. While we also recovered a predicted homolog of the luciferase of the firefly *Luciola*, this is also in the much larger 4-coumarate-CoA ligase gene family. The recovery of a luciferase, and perhaps too the CoA ligase with strong sequence similarity to a firefly luciferase, corroborates prior suppositions regarding the biochemical process of light emission in *O*. *enopla* [31] and casts doubt on the role of any photoproteins in bioluminescing *O*. *enopla*. Recently, Mehr et al. [42] recovered a photoprotein-like transcript in *Hermodice carunculata*, but without evidence for bioluminescent activity for such a protein from an amphinomid polychaete not known to bioluminesce. Other highly similar sequences (at 1e^-107^) to those reported by Mehr et al. [42] also exist among the transcripts of the non-bioluminescent *Platynereis dumerilii* (*i*.*e*., GenBank Acc No JZ443006 [EST: KN-1127-B-26_E04_SP6] and JZ462998 [EST: KN-1127_B-50_C10_SP6]).

Multiple paralogs of the *Odontosyllis-*luciferase gene family were recovered from each of three *O*. *enopla* transcriptomes generated in this study. We also located two unreported paralogs of the luciferase gene in the published *O*. *undecimdonta* transcriptome. Fig 3 illustrates that the four previously reported isoforms of the *O*. *undecimdonta* luciferase gene group together with our de-novo predicted *O*. *undecimdonta* DN31989 transcript. The predicted orthologous luciferase genes from *O*. *enopla* group sister to those and cluster among other *O*. *undecimdonta* predicted transcripts. Additional paralogs of this *Odontosyllis* luciferase gene family form six distinct clusters, most of which were recovered from all three individuals of *O*. *enopla*. While these results might indicate that *O*. *undecimdonta* has fewer members of this particular gene family compared to *O*. *enopla*, a difference in the physiological state of worms may be explanatory. Our individuals of *O*. *enopla* were captured and preserved while actively luminescing. Individuals of *O*. *undecimdonta* were captured "At dusk, *Odontosyllis* worms were attracted to a handheld light at the surface and collected with a hand dip net. Worms were individually preserved in Invitrogen RNAlater or lyophilized for later analysis” (Schultz et al. [44]).

The specifics of which gene expression profiles vary with lunar cycles differs phylogenetically [51]. Transcripts from *O*. *enopla* comprise most of those loci already known to be responsible for entrainment and modification of circadian and circalunar periodicity in the *P*. *dumerilii* polychaete model [50]. Specifically, in *P*. *dumerilii*, each of *clock*, *period*, *pdp1* and *timeless* circadian oscillator loci are modified by the worm’s endogenous circalunar clock, while expression levels of *bmal*, *tr-cry*, *vrille*, and *timeout* are not. The cryptochrome *l-cry* has already been demonstrated to be involved in forebrain blue-light direct photo reception whereas *tr-cry* orthologs function in a core circadian clock positive/negative transcriptional loop [50].

*Odontosyllis enopla* and *O*. *luminosa* exhibit extremely tight circalunar circadian behavior with bioluminescent mating swarms being predictable to the minute [8, 29]. Should *O*. *enopla* be successfully kept in laboratory culture for extended periods, transcripts identified here should serve to shed new light on the level of control exerted on bioluminescence by circadian clock gene systems.

During the breeding period, female eyes become enlarged and heavily pigmented with a carotenoid (putatively a rhodopsin system). Several genes putatively corresponding to these epitoky-related changes of the eyes were recovered from *O*. *enopla* transcripts. Of particular interest is a predicted homolog of a phototransduction gene (354 amino acids in length) that is similar to that found within the retinal transcriptome of the sabellid polychaete *Acromegalomma interruptum* (GenBank Acc No ASQ43263). Optic-related phototransduction proteins are associated with ciliary photoreceptors, the latter of which hyperpolarize in response to illumination [52]. This process may be important during mating to coordinate spatially with males that are swimming rapidly toward glowing females while emitting short flashes of light in advance of releasing their own gametes. Other putative eye-related genes included: 1) a retinitis pigmentosa GTPase regulator (761 aa), which may help maintain photoreceptors by regulating the formation and function of cilia [53]; 2) a retinol dehydrogenase (retinaldehyde reductase) (327 aa) and epidermal retinol dehydrogenase 2-like isoform (306 aa), which are expressed in retinal pigment epithelium and help generate the polyene chromophore retinaldehyde (also known as retinal or vitamin A [54]; and 3) a carotenoid isomerooxygenase-like gene (529 aa) that is putatively involved in the synthesis of retinal from dietary caroteoids [55]. The presence of predicted homologs of retinol dehydrogenase and carotenoid isomerooxygenase is important as opsin-bound retinal is the chemical basis of vision. We also recovered a predicted homolog of a Class A rhodopsin-like G-protein coupled receptor (319 aa), which may function in light receptors. However, rhodopsin-like GPCRs can also function as hormones, which, if confirmed, would suggest that interindividual communication is also being conducted chemically via sex pheromones. We also recovered a predicted homolog of a photoreceptor-specific-like isoform of phosphatidate cytidylyltransferase (442 aa), which may be involved in the signal transduction mechanism of retina and neural cells, and a retinal rod rhodopsin-sensitive cGMP 3',5'-cyclic phosphodiesterase subunit delta gene (147 aa), which is involved in visual perception.

The nephridial system is also modified to store and release gametes and associated secretions during the breeding period. Several genes putatively corresponding to epitoky-related changes of the nephridia were also recovered from *O*. *enopla* transcripts. These included predicted homologs of a nephrocystin-3 (347 aa), a nephrocystin-3-like gene (437 aa), and a sex peptide receptor-like gene (178 aa). Nephrocystin-3 is required for normal ciliary development and function but is also involved in the development and morphogenesis of human kidneys. Nephridia are synonymous with the excretory organs of higher organisms (*i*.*e*., kidneys). During epitoky, nephrocystin-3 may help modify the cilia-lined tubules of the nephridia to store and release gametes, as well as release fluids that provide nutrition and support for the gametes. According to [56], sex peptide receptor (SPR) mediates the release of stored sperm, receptivity, egg production, and egg release in female *Drosophila melanogaster*. We hypothesize that SRP may have a similar function in *O*. *enopla* with regard to egg release during bioluminescent mating swarms.

Future studies should conduct a control experiment (i.e., analyzing non-mating worms from another day, males, etc.) to determine if the transcripts recovered during a bioluminescent mating swarm are specifically involved in the functions and/or processes we are hypothesizing herein, or whether they are expressed at all times in relation to general functions.

## Conclusions

From each of three actively luminescing female *O*. *enopla*, we recovered a complete open reading frame matching a known and recently discovered *Odontosyllis*-specific luciferase gene family. We also recovered a CoA ligase that shows strong sequence similarity to a firefly luciferase. These findings corroborate prior suppositions regarding the biochemical process of light emission in *O*. *enopla* to the exclusion of photoproteins. Given the large suite and diversity of luciferase transcripts recovered from each of the three *O*. *enopla* transcriptomes, this species might serve as an ideal model organism to study the evolution and selection pressures apparent in this new luciferase gene family. The transcriptome also yielded a number of genes putatively related to circadian or circalunar periodicity, as well as epitoky-related changes of the eyes and nephridial system, all of which are important for successful breeding. The origins and evolutionary history of this and other luciferases will ultimately require a more complete understanding of the range of monooxygenases found among the vast diversity of taxa that lie between fireflies and syllid polychaetes.

## Supporting information

S1 Fig FASTA file of translated nucleotide sequences of four *Odontosyllis enopla* genes putatively associated with periodicity. This includes predicted homologs of *clock* (691 amino acids in length), *bmal*1 (565 aa), *period* (1,652 aa) and *timeless* (1,349 aa).
(FASTA)

S2 Fig FASTA file of translated nucleotide sequences of two putative *Odontosyllis enopla* photosensitive cryptochrome transcripts. This includes predicted homologs of a light-receptive cryptochrome (*l-cry*; 134 amino acids in length) and a transcription factor with a putative circadian oscillator component (*tr-cry*; 591 aa).
(FASTA)

S3 Fig FASTA file of translated nucleotide sequences of putative eye-related genes located within the *Odontosyllis enopla* transcriptome. See Table 1 for the lengths (in amino acids) of all predicted homologs.
(FASTA)

S4 Fig FASTA file of translated nucleotide sequences of putative nephridia-related genes located within the *Odontosyllis enopla* transcriptome. See Table 2 for the lengths (in amino acids) of all predicted homologs.
(FASTA)

## References

[1] Crawshay LR. Possible bearing of a luminous syllid on the question of the landfall of Columbus. Nature. 1935 10 5;136(3440):559–60.

[2] Markert RE, Markert BJ, Vertrees NJ. Lunar periodicity in spawning and luminescence in *Odontosyllis enopla*. Ecology. 1961 4 1;42(2):414–5.

[3] Fischer A, Fischer U. On the life-style and life-cycle of the luminescent polychaete *Odontosyllis enopla* (Annelida: Polychaeta). Invertebrate Biology. 1995 7 1:236–47.

[4] Franke HD. Reproduction of the Syllidae (Annelida: Polychaeta). Hydrobiologia. 1999 5 1;402:39–55.

[5] Galloway TW, Welch PS. Studies on a phosphorescent Bermudan annelid, *Odontosyllis enopla* Verrill. Transactions of the American Microscopical Society. 1911 1 1;30(1):13–39.

[6] Tsuji FI, Hill E. Repetitive cycles of bioluminescence and spawning in the polychaete, *Odontosyllis phosphorea*. The Biological Bulletin. 1983 10;165(2):444–9. 10.2307/1541210 28368224

[7] Wolken JJ, Florida RG. The eye structure of the bioluminescent fireworm of Bermuda, *Odontosyllis enopla*. The Biological Bulletin. 1984 2;166(1):260–8.

[8] Gaston GR, Hall J. Lunar periodicity and bioluminescence of swarming *Odontosyllis luminosa* (Polychaeta: Syllidae) in Belize. Gulf and Caribbean Research. 2000;12(1):47–51.

[9] Deheyn DD, Latz MI. Internal and secreted bioluminescence of the marine polychaete *Odontosyllis phosphorea* (Syllidae). Invertebrate biology. 2009 2 1;128(1):31–45.

[10] Daly JM. Reversible epitoky in the life history of the polychaete *Odontosyllis polycera* (Schmarda 1861). Journal of the Marine Biological Association of the United Kingdom. 1975 5;55(2):327–44.

[11] San Martín G, Aguado MT. Family Syllidae. Phyllodocida: Nereidiformia. Handbook of Zoology, Annelida A Natural History of the Phyla of the Animal Kingdom. Verlag Walter der Gruyter GmbH & Co 2014.

[12] San Martín G, Worsfold TM. Guide and keys for the identification of Syllidae (Annelida, Phyllodocida) from the British Isles (reported and expected species). ZooKeys. 2015(488):1 10.3897/zookeys.488.9061 25878521 PMC4389122

[13] Aguado MT, San Martín G, Siddall ME. Systematics and evolution of syllids (Annelida, Syllidae). Cladistics. 2012 6 1;28(3):234–50. 10.1111/j.1096-0031.2011.00377.x 34872192

[14] Aguado MT, Nygren A, Siddall ME. Phylogeny of Syllidae (Polychaeta) based on combined molecular analysis of nuclear and mitochondrial genes. Cladistics. 2007 12 1;23(6):552–64. 10.1111/j.1096-0031.2007.00163.x 34905869

[15] Zörner SA, Fischer A. The spatial pattern of bioluminescent flashes in the polychaete *Eusyllis blomstrandi* (Annelida). Helgoland Marine Research. 2007 3 1;61(1):55–66.

[16] Bassot JM. Sites actifs et facilitation dans trois systèmes bioluminescents. Arch Zool Exp Gen. 1979;120:5–24.

[17] Ellis EA, Oakley TH. High rates of species accumulation in animals with bioluminescent courtship displays. Current Biology. 2016 7 25;26(14):1916–21. 10.1016/j.cub.2016.05.043 27345160

[18] Pleijel F. Syllidae Grube, 1850 In: Rouse GW & Pleijel F (Eds), Polychaetes 2001 10 11 (pp.102–105). Oxford University Press, New York.

[19] Plyuscheva M, Martin D. On the morphology of elytra as luminescent organs in scale-worms (Polychaeta, Polynoidae). Zoosymposia. 2009 8 31;2(1):379–89.

[20] Pes O, Midlik A, Schlaghamersky J, Zitnan M, Taborsky P. A study on bioluminescence and photoluminescence in the earthworm *Eisenia lucens*. Photochemical & Photobiological Sciences. 2016;15(2):175–80. 26786938 10.1039/c5pp00412h

[21] Verdes A, Gruber DF. Glowing Worms: Biological, Chemical, and Functional Diversity of Bioluminescent Annelids. Integrative and Comparative Biology. 2017 7 1; 57(1):18–32. 10.1093/icb/icx017 28582579

[22] Huber ME, Arneson CA, Widder EA. Extremely blue bioluminescence in the polychaete *Polycirrus perplexus* (Terebellidae). Bulletin of Marine Science. 1989 5 1;44(3):1236–9.

[23] Nishi E, Arai H, Sasanuma SI. A new species of *Chaetopterus* (Polychaeta: Chaetopteridae) from off Tokyo Bay, Central Japan, with comments on its Bioluminescence. Actinia. 2000 3 1;13: 1–12.

[24] Gouveneaux A, Flood PR, Erichsen ES, Olsson C, Lindström J, Mallefet J. Morphology and fluorescence of the parapodial light glands in *Tomopteris helgolandica* and allies (Phyllodocida: Tomopteridae). Zoologischer Anzeiger-A Journal of Comparative Zoology. 2016 8 10.

[25] Osborn KJ, Haddock SH, Pleijel F, Madin LP, Rouse GW. Deep-sea, swimming worms with luminescent “bombs”. Science. 2009 8 21;325(5943):964–. 10.1126/science.1172488 19696343

[26] Bassot JM, Nicolas MT. Bioluminescence in scale-worm photosomes: the photoprotein polynoidin is specific for the detection of superoxide radicals. Histochemistry and cell biology. 1995 9 1;104(3):199–210. 8542446 10.1007/BF01835153

[27] Shimomura O. Bioluminescence: chemical principles and methods. World Scientific; 2006.

[28] Goodrich ES. Memoirs: Notes on *Odontosyllis*. Journal of Cell Science. 1933 10 1;2(302):319–29.

[29] Huntsman AG. *Odontosyllis* at Bermuda and lunar periodicity. Journal of the Fisheries Board of Canada. 1948 6 1;7(6):363–9.

[30] Wilkens LA, Wolken JJ. Electroretinograms from *Odontosyllis enopla* (Polychaeta; Syllidae): initial observations on the visual system of the bioluminescent fireworm of Bermuda. Marine & Freshwater Behaviour & Phy. 1981 1 1;8(1):55–66.

[31] Harvey EN. Bioluminescence. Academic Press; 1952.

[32] Deheyn DD. Bioluminescence characteristics of the marine worm *Odontosyllis phosphorea*. In LUMINESCENCE 2006 9 1 (Vol. 21, No. 5, pp. 274–274). Commerce Place, 350 Main St, Malden 02148, MA USA: Wiley-Blackwell.

[33] Erdman DS. Lunar periodicity in the swarming of luminescent worms, *Odontosyllis octodentata* Treadwell (Annelida) off La Parguera, PR. Caribbean Journal of Science. 1965;5:103–7.

[34] Inoue S, Okada K, Tanino H, Kakoi H, Ohnishi Y, Horii N. New lumazines from the marine polychaete, *Odontosyllis undecimdonta*. Chemistry Letters. 1991 4;20(4):563–4.

[35] Sato N, Fukuya S. Studies on pyrazines. Part 37. 1 Synthesis of 6-propionylpteridine-2, 4 (1 H, 3 H)-dione and its 1-and/or 3-methyl derivatives from marine natural products. Journal of the Chemical Society, Perkin Transactions 1. 2000(1):89–95.

[36] Zhang Y, Sun J, Chen C, Watanabe HK, Feng D, Zhang Y, et al Adaptation and evolution of deep-sea scale worms (Annelida: Polynoidae): insights from transcriptome comparison with a shallow-water species. Scientific Reports. 2017; 7:46205 10.1038/srep46205 28397791 PMC5387418

[37] Weigert A, Conrad H, Meyer M, Birgit N, Detlev A, Bernhard S, et al Illuminating the base of the annelid tree using transcriptomics. Mol. Biol. Evol. 2014;31,1391–1401. 10.1093/molbev/msu080 24567512

[38] Heikkinen LK, Kesäniemi JE, Knott KE. De novo transcriptome assembly and developmental mode specific gene expression of *Pygospio elegans*. Evolution & Development. 2017;19:205–217. 28869352 10.1111/ede.12230

[39] Kvist S, Brugler MR, Goh TG, Giribet G, Siddall ME. Pyrosequencing the salivary transcriptome of *Haemadipsa interrupta* (Annelida: Clitellata: Haemadipsidae): anticoagulant diversity and insight into the evolution of anticoagulation capabilities in leeches. Invertebrate biology. 2014 3 1;133(1):74–98.

[40] Novo M, Riesgo A, Fernández-Guerra A, Giribet G. Pheromone Evolution, Reproductive Genes, and Comparative Transcriptomics in Mediterranean Earthworms (Annelida, Oligochaeta, Hormogastridae). Mol Biol Evol. 2013 7;30(7):1614–29. 10.1093/molbev/mst074 23596327

[41] Richter S, Helm C, Meunier FA, Hering L, Campbell LI, Drukewitz SH, et al Comparative analyses of glycerotoxin expression unveil a novel structural organization of the bloodworm venom system. BMC Evolutionary Biology. 2017;17:64 10.1186/s12862-017-0904-4 28259138 PMC5336659

[42] Mehr S, Verdes A, DeSalle R, Sparks J, Pieribone V, Gruber DF. Transcriptome sequencing and annotation of the polychaete *Hermodice carunculata* (Annelida, Amphinomidae). BMC genomics. 2015 12 1;16(1):445. 26059236 10.1186/s12864-015-1565-6 PMC4462082

[43] Ponz G, Bleidorn C, Aguado MT. Expression of vasa, piwi, and nanos during gametogenesis in *Typosyllis antoni* (Annelida, Syllidae). Evolution and development. *Submitted*. 10.1111/ede.12263 30094969

[44] Schultz DT, Kotlobay AA, Ziganshin R, Bannikov A, Markina NM, Chepurnyh TV, et al Luciferase of the Japanese syllid polychaete *Odontosyllis undecimdonta*. Biochemical and biophysical research communications. 2018 7 20;502(3):318–323. 10.1016/j.bbrc.2018.05.135 29792858

[45] Roda A, Pasini P, Mirasoli M, Michelini E, Guardigli M. Biotechnological applications of bioluminescence and chemiluminescence. TRENDS in Biotechnology. 2004 6 30;22(6):295–303. 10.1016/j.tibtech.2004.03.011 15158059

[46] Bolger AM, Lohse M, Usadel B. Trimmomatic: a flexible trimmer for Illumina sequence data. Bioinformatics. 2014 4 1;30(15):2114–20. 10.1093/bioinformatics/btu170 24695404 PMC4103590

[47] Parra G, Bradnam K, Korf I. CEGMA: a pipeline to accurately annotate core genes in eukaryotic genomes. Bioinformatics. 2007 3 1;23(9):1061–7. 10.1093/bioinformatics/btm071 17332020

[48] Marchler-Bauer A, Bo Y, Han L, He J, Lanczycki CJ, Lu S, et al CDD/SPARCLE: functional classification of proteins via subfamily domain architectures. Nucleic acids research. 2016 11 28;45(D1):D200–3. 10.1093/nar/gkw1129 27899674 PMC5210587

[49] Källberg M, Wang H, Wang S, Peng J, Wang Z, Lu H, et al Template-based protein structure modeling using the RaptorX web server. Nature protocols. 2012 8 1;7(8):1511–22. 10.1038/nprot.2012.085 22814390 PMC4730388

[50] Zantke J, Ishikawa-Fujiwara T, Arboleda E, Lohs C, Schipany K, Hallay N, et al Circadian and circalunar clock interactions in a marine annelid. Cell reports. 2013 10 17;5(1):99–113. 10.1016/j.celrep.2013.08.031 24075994 PMC3913041

[51] Brady AK, Willis BL, Harder LD, Vize PD. Lunar phase modulates circadian gene expression cycles in the broadcast spawning coral *Acropora millepora*. The Biological Bulletin. 2016 4;230(2):130–42. 10.1086/BBLv230n2p130 27132135

[52] Bok MJ, Porter ML, Nilsson DE. Phototransduction in fan worm radiolar eyes. Current Biology. 2017 7 24;27(14):R698–9. 10.1016/j.cub.2017.05.093 28743013

[53] Gakovic M, Shu X, Kasioulis I, Carpanini S, Moraga I, Wright AF. The role of RPGR in cilia formation and actin stability. Human molecular genetics. 2011 9 20;20(24):4840–50. 10.1093/hmg/ddr423 21933838

[54] Simon A, Romert A, Gustafson AL, McCaffery JM, Eriksson U. Intracellular localization and membrane topology of 11-cis retinol dehydrogenase in the retinal pigment epithelium suggest a compartmentalized synthesis of 11-cis retinaldehyde. Journal of Cell Science. 1999 2 15;112(4):549–58. 9914166 10.1242/jcs.112.4.549

[55] Oberhauser V, Voolstra O, Bangert A, Von Lintig J, Vogt K. NinaB combines carotenoid oxygenase and retinoid isomerase activity in a single polypeptide. Proceedings of the National Academy of Sciences. 2008 12 2;105(48):19000–5. 10.1073/pnas.0807805105 PMC2596218 19020100

[56] Avila FW, Mattei AL, Wolfner MF. Sex peptide receptor is required for the release of stored sperm by mated *Drosophila melanogaste*r females. Journal of insect physiology. 2015 5 31;76:1–6. 10.1016/j.jinsphys.2015.03.006 25783955 PMC4430431

